# Repercussions of perineal repair using surgical glue or suture thread on postpartum outcomes: A controlled randomized clinical trial in São Paulo, Brazil

**DOI:** 10.18332/ejm/191248

**Published:** 2024-08-23

**Authors:** Wesllanny S. Brunelli, Adriana Caroci Becker, Marlise O. P. Lima, Sheyla G. Oliveira, Angela M. Ochiai, Lucca Caroci, Natalucia M. D. Araújo, Maria L. Riesco

**Affiliations:** 1 Maternal and Child Department, School of Nursing, São Paulo University, São Paulo, Brazil; 2 Midwifery Department, School of Arts, Sciences and Humanities, São Paulo University, São Paulo, Brazil; 3 Doctor Moysés Deutsch Municipal Hospital, São Paulo, Brazil; 4 Healthcare Management Program, St. Francis College, New York, United States

**Keywords:** postpartum period, urinary incontinence, natural childbirth, perineum, tissue adhesives, pelvic floor, labor

## Abstract

**INTRODUCTION:**

The type of perineal repair can have significant long-term effects on various functions in a woman's postpartum life. The aim was to compare urinary incontinence (UI), women's satisfaction, pelvic floor muscle strength (PFMS), and sexual function according to the type of perineal repair (surgical glue or suture thread) during the first eight months after normal childbirth.

**METHODS:**

A controlled randomized clinical trial of 133 primiparous women undergoing perineal repair during birth with surgical glue or sutures, evaluated during labor and monitored up to 8 months postpartum, from March 2017 to September 2018, in the city in São Paulo, Bazil. Descriptive and inferential analyses were carried out.

**RESULTS:**

A total of 133 women were included in the study, 111 (83.5%) were assessed between 10 to 20 days postpartum, 121 (91.0%) between 50 to 70 days, and 54 (40.6%) between 6 to 8 months. There were no significant differences for UI concerning the type of repair; however, there was a significant difference concerning the postpartum period (p=0.031), with a higher prevalence at two months. Most women reported satisfaction, with the highest levels reported two months after birth (p=0.019). For PFMS, the mean of the glue and suture groups were 32.4 cmH_2_O and 27.4 cmH_2_O, but not significant. Women in the glue group showed higher mean values in all sexual function domains but without significance.

**CONCLUSIONS:**

Surgical glue showed good aesthetic and functional results in the perineum at eight months postpartum.

## INTRODUCTION

In vaginal births, possible damage to the pelvic innervation and tears in the muscles lead to the weakening of the supporting tissues of the pelvic diaphragm, which can trigger the presence and increase of pelvic floor disorders, such as pelvic organ prolapse, reduction of the pelvic floor muscle strength (PFMS), urinary incontinence (UI) and sexual dysfunctions^1,2^. These disorders negatively affect women’s quality of life through limitations in physical, social, and sexual activities, as well as psychological distress^3^.

In the clinical scenario, the concept of sexual function covers sexuality in different aspects, such as sexual activity, desire, and satisfaction of women. Therefore, the timely identification of risk factors for the occurrence of pelvic floor dysfunctions, especially perineal trauma in vaginal birth, is of great clinical importance to prevent and treat diseases and promote maternal rehabilitation^1,4-8^.

Therefore, when perineal trauma is present during vaginal birth, the repair of damaged tissues should be a concern for everyone who attends the birth because the severity of the perineal trauma, the professional’s skill in surgical repair, the type of material used, and the perineal repair technique applied, can produce or increase maternal morbidity in the short- and long-term^9^.

The continuous suture technique with polyglactin 910 (Vicryl Rapide^®^) thread has been considered the gold standard for perineal repair. It reduces perineal pain, improves perineal trauma healing, and increases women’s satisfaction with perineal repair^10^.

Recently, preliminary studies investigated perineal repair with surgical glues, obtaining favorable results in their application, such as less perineal pain reported by women, greater women’s satisfaction with perineal repair, shorter application time, and adequate perineal healing, not inferior to traditional suturing in terms of aesthetic and functional results^11,12^. Another study used non-surgical glue (ethyl 2-cyanoacrylate) to repair first-degree perineal laceration and found similar results^13^. One more advantage presented by glue is that its use is easy to learn, given that an untrained professional can master the technique after up to 5 procedures^11^.

In order to minimize perineal damage during normal childbirth and the consequent need for more invasive interventions in its repair, a systematic review points to the adoption of techniques that can reduce tears in first-time mothers and anal sphincter damage, such as perineal massage at the end of the third trimester; perineal support and massage and warm compresses during the second stage of labor^5^. For hemostatic in small first and second-degree without anatomical distortion, conservative care is indicated to decrease pain and reduce analgesia and dyspareunia. Surgical glue may be a less invasive option for minor hemostatic lesions with anatomical disruption^6,14^. Also, this could lead to reduced intervention time and improve healing outcomes.

However, further research is needed to fully understand the long-term impacts on perineal health and sexual function^7^. The present study compared perineal repair methods to mitigate a significant health problem in women’s lives.

With the inclusion of surgical glue as an option for perineal repair and the impact that pelvic disorders, aggravated by perineal trauma, exert on maternal health, it becomes necessary to assess the results of applying surgical glue in perineal repair for a more extended period to verify whether there is an improvement in the postpartum perineal outcomes compared to standard care. Therefore, the aim was to compare UI, women’s satisfaction, PFMS, and sexual function according to the type of perineal repair (surgical glue or suture thread) during the first eight months after normal childbirth.

## METHODS

### Study design and setting

Open four-parallel groups (1:1:1:1) randomized controlled trial (RCT) was conducted in six stages. Stages 1 to 4 have already been reported elsewhere^7,15^. The data from stages 4, 5, and 6 have been presented. The study was conducted alongside the Normal Birth Center (NBC) in the São Paulo, Brazil, metropolitan region. The center was chosen because it is an NBC, making it a prime location for research on this subject. The birth center exclusively cares for low-risk pregnant women. The nurse-midwives and midwives assist women with normal births, offering non-pharmacological pain relief methods and, if necessary, performing the perineal trauma sutures with local anesthesia. In Brazil, they obtain their degrees directly through entrance to midwifery or post-degree specialization in nursing midwifery. Their scope of practice involves caring for women in public health women’s programs, pregnancy, normal labor and birth, and the care of healthy newborns.

### Participants and sample size

The participants were women with first- or second-degree spontaneous perineal tears or episiotomy. After birth, they were allocated into four groups. Experimental Group 1: women with first-degree tears to be repaired by Glubran-2^®^ surgical glue; and Experimental Group 2: women with second-degree tears or episiotomy to be repaired by Glubran-2^®^ surgical glue. Control Group 1: women with first-degree tears to be repaired by Vicryl Rapid^®^ suture thread; and Control Group 2: women with second-degree tears or episiotomy to be repaired by Vicryl Rapid^®^ suture thread.

The sample was designed to detect a minimum significant difference of 2 points in pain score between the two perineal repair methods. To this end, an *a priori* standard deviation of 3 points, an alpha error of 5%, and a power of 80% were considered, resulting in a minimum sample of 35 women in each group. Thus, the sample consisted of 140 women: 70 allocated to the experimental groups 1 (n=35) and 2 (n=35) and another 70 to the control groups 1 (n=35) and 2 (n=35). The statistical software Bioestat^®^ 5.3 was used to estimate the sample.

### Inclusion and exclusion criteria

Inclusion criteria were: primiparous; having up to 6 cm of cervical dilation at the time the woman was invited to participate in the research; not using steroid substances; not presenting leukorrhea or any signs of infection at the repair site; no difficulty understanding the Portuguese language or in communication; and accepting to be subjected to perineal repair methods with surgical glue or suture thread. The women included in the study underwent vaginal birth with first- and second-degree spontaneous perineal tears or episiotomy. Women were excluded if they did not continue in the study or did not return to the study stages.

### Randomization

The order in which participants entered each group was randomized using a table of random numbers generated electronically using the Statistical Package for the Social Sciences (SPSS). Opaque envelopes containing the sequence in the glue or suture group were used. One of the birth attendants opened these envelopes at the time of the perineal repair, maintaining the integrity of the study and ensuring that the researchers were not involved in the allocation process.

Each participant was informed which group they were assigned to, and the researchers explained the procedure according to the group. The suture group was informed about local anesthesia before suturing. Because of the nature of the study, it was not possible to blind the participants and the researchers. Women who requested to be excluded from the study were replaced by others, following the randomization table.

### Interventions and materials

The interventions involved using Glubran-2^®^ and Vicryl Rapid^®^ suture thread to repair episiotomies and first- and second-degree perineal lacerations. The Glubran-2^®^ is a synthetic surgical glue based on N-Butyl-2 cyanoacrylate, manufactured by GEM S.r.l in Italy. It is a medical-surgical product for internal and external use under European Directive 93/42/EEC requirements. The glue polymerization time varies depending on the type of tissue the glue comes into contact with, the nature of the fluids present, and the amount of product applied^16^.

Vicryl Rapid^®^ suture thread, whose technical name is polyglactin 910, is produced by Ethicon – Johnson & Johnson. It comprises a glycolide of 90% and a lactide of 10%, covered by 50% polyglactin 370 and 50% calcium stearate. It is braided, colorless, and sterilized with Cobalt-60. The *in vivo* tensile strength is 100% on the first day, 81% on the third, 57% on the fifth, 53% on the seventh, and 0% after 14 days. Complete absorption occurs in approximately 35 days by hydrolysis^12,15^.

### Outcomes and variables

The primary outcomes were the occurrence and intensity of perineal pain, the woman’s level of satisfaction with the repair, perineal repair time, perineum healing process and aesthetics, complications related to perineal repair in the postpartum period, need for suturing with thread in the experimental group due to failed perineal repair using glue, up to 20 days after birth, which have already been published^7,15^. None of the postpartum women participants in this research experienced dehiscence.

This part of the study presents data relating to secondary outcomes: UI, women’s satisfaction with perineal repair, PFMS, and sexual function according to the type of perineal repair from 2 to 8 months after birth.

Other variables considered were maternal sociodemographic and clinical characteristics (age, ethnicity, education level, occupation, marital status, previous disease, body index mass, gestational age, and perineal conditions during birth).

### Data sources and measurements

During the UI evaluation, the participants were asked about their previous or current UI, considering the involuntary urine loss, amount, and time. As far as the researchers know, no validated translated tool was available to evaluate postpartum UI at the time of this study.

The satisfaction with perineal trauma repair was assessed using a five-point scale. Women were asked to observe the perineal repair region and answer the question: ‘How satisfied are you with the perineal repair?’ with response options: very dissatisfied, dissatisfied, indifferent, satisfied, and very satisfied. UI and satisfaction were evaluated at 2, 6, and 8 months after birth.

Sexual function was assessed using the Feminine Sexual Function Index (FSFI) instrument. The FSFI, translated from English and validated in Brazil, includes 19 questions that assess female sexual responses to sexual desire, arousal, vaginal lubrication, orgasm, sexual satisfaction, and perineal pain. Each answer is assigned a value and weight, and a score between 2 and 36 points is obtained at the end. The instrument was applied in the face-to-face interview modality^17^.

PFMS was measured with a Peritron^®^ perineometer. The Peritron^®^ device, from Laborie, Canada, consists of a silicone probe with a length of 8 cm and a diameter of 3 cm. A sensor is connected to the probe, which records the pressure applied on a scale from 0.1 to 300 cmH_2_O when linked to a handheld microprocessor. When introducing the probe into the woman’s vaginal canal and when the pelvic floor muscles pressure the probe, the sensor records the pressure value of the muscle contractions exerted by the woman. In order to measure PFMS, the technique followed was the one presented by studies previously carried out in the midwifery and obstetrics field^7,15,18^. Sexual function and PFMS were evaluated at 6 and 8 months postpartum.

### Procedures before data collection

The investigators, who are nurse midwives, were previously trained and sought to minimize information bias. They participated in the data collection by performing perineal trauma repair with surgical glue or suture thread. They were previously trained in applying surgical glue to beef tongues and other cuts of beef, such as palette tips and hams. They were able to observe the effectiveness of the adhesive because even when the bovine tissues were wet from the saline solution applied or from blood, the adhesive proved to be highly resistant to the tension of these tissues. These procedures were videotaped.

It is also worth mentioning that a pilot study with 19 women from each group was carried out before initiating the data collection to make the necessary adjustments to the project, especially to the interventions^18^. The researchers already had experience using the continuous perineal suture technique with thread, as they used it to teach and assist women during childbirth.

### Data collection

The eligible participants were 140 primiparous women who underwent vaginal birth with first- and second-degree laceration or episiotomy and who satisfied the inclusion criteria. The study included them for perineal repair with Glubran-2^®^ surgical glue (experimental groups) or Vicryl Rapid^®^ suture thread (control groups). They were evaluated during labor and monitored up to 8 months after birth, from March 2017 to September 2018, by the six following stages:

- Stages 1, 2, 3, and 4: during labor and up to 2 hours after the perineal repair procedure, from 12 to 24 hours, from 36 to 48 hours, and from 10 to 20 days postpartum, respectively. The primary outcomes were assessed (perineal pain, woman’s satisfaction with the repair, perineal repair time, perineum healing process and aesthetics, complications related to perineal repair)^7,15^. At stage 4, UI was also assessed.
- Stage 5: From 50 to 70 days postpartum. The UI and women’s satisfaction were assessed.
- Stage 6: From 6 to 8 months postpartum. The UI, women’s satisfaction, PFMS, and sexual function were assessed.

**Figure 1:**
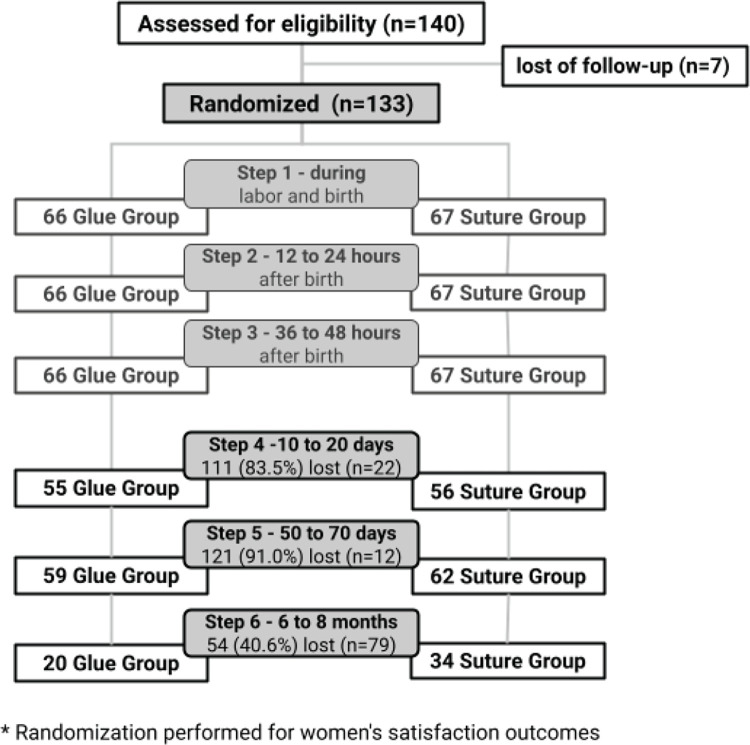
Flowchart of primiparous women undergoing perineal repair during birth with surgical glue or sutures, RCT follow-up, São Paulo, Brazil, 2017–2018 (N=133)

Stages 1, 2, and 3 were conducted during the hospitalization at the NBC by interview and clinical examination. Stages 4 and 5 were conducted after discharge, mainly by face-to-face or phone interview. The phone interview was used if the woman missed two consecutive appointments at the maternity clinic or did not agree to a home visit. It is important to note that the home visit was conducted solely to collect data. The phone interview lasted approximately five minutes. In order to assess her satisfaction with the perineal repair, the woman was asked to use a mirror to visualize the repair area over the phone and then report her satisfaction. Stage 6 was conducted exclusively face-to-face, through interviews and clinical examinations. Seven eligible women did not participate in any postpartum assessments after discharge. Thus, 133 women (66 with perineal repair by glue and 67 by suture thread) were followed up in stages 4, 5, and 6 (Figure 1).

### Data analysis

Qualitative variables are presented as frequencies and percentages, while quantitative variables are presented using appropriate descriptive statistics (i.e. mean, median, SD, 95% CI, min, and max). The chi-squared test was used to compare categorical variables. The Mann–Whitney U test and the t-test for independent samples were used to analyze continuous variables, with the choice of test based on the assumptions made for each variable. Residual normality and homoscedasticity were assessed in each case to determine the most suitable test. The Mann–Whitney U test was used under the same assumptions as the Student’s t-test, except for residual normality. The binomial and cumulative binomial generalized linear mixed model (GLMM) for repeated measures was used to assess three effects: types of perineal repair, stages, and the interaction between types of repair and stages. The significance level was set at p<0.05.

### Ethical considerations

The study, approved by the Research Ethics Committee of the School of Arts, Sciences, and Humanities - CAAE 44832615.1.0000.5390 and the Municipal Health Council of Itapecerica of the Serra, followed all the determinations outlined in Resolution 466/2012 of the National Health Council^19^, ensuring the protection of the human rights of all research participants. The trial was registered in the Brazilian Registry of Clinical Trials (UTN code: U1111-1184-2507; RBR-2q5wy8o); date of registration 01/25/2018; www.ensaiosclinicos.gov.br/rg/RBR-2q5wy8/. The women included in the study read and signed the Informed Consent Form, thereby participating in a transparent and informed research process. All the project costs were funded by research agencies (National Council for Scientific and Technological Development and Research Support Foundation of the State of São Paulo) and not by the materials’ manufacturers.

## RESULTS

Among all 133 women included in the sample, data were collected from 111 (83.5%) of them between 10 and 20 days after birth, from 121 (91.0%) between 50 and 70 days, and from 54 (40.6%) between 6 and 8 months postpartum. Considering these stages (stages 4, 5, and 6), 286 evaluations were performed: 189 at the maternity clinic, 69 at the women’s homes, and 28 by telephone. The reasons for the follow-up losses were that the participant did not attend the scheduled return appointment and refused to receive a home visit, the researchers could not access the participant’s house, and the researchers lost contact with the participant.

The women’s sociodemographic and clinical characteristics are presented in Table 1, which shows the homogeneity of both groups for all variables. Most women identified themselves as mixed-race, followed by white, had at least completed high school; house chores were the occupation of most participants; most of them lived with a partner and did not have previous disease. Regarding the type of perineal trauma among the follow-up participants, first-degree laceration was the most common, occurring in 68.2% of the women in the glue group and 55.2% in the suture group. Episiotomy had the lowest incidence, with 3% in the glue group and 9% in the suture group. The groups were also similar in terms of maternal and gestational and body mass index.

**Table 1:** Sociodemographic and clinical characteristics of primiparous women undergoing perineal repair during birth with surgical glue or sutures, according to the type of perineal repair, RCT follow-up, São Paulo, Brazil, 2017–2018 (N=133)

*Characteristics*	*Perineal repair*				*p*
	*Glue (N=66)*		*Suture (N=67)*		
	*n*	*%*	*n*	*%*
**Ethnicity**					
Mixed-race	38	57.6	31	46.2	0.111*
White	21	31.8	20	29.9	
Black	5	7.6	15	22.4	
Asian	2	3.0	1	1.5	
**Education level**					
Incomplete elementary school	4	6.1	3	4.5	0.477*
Complete elementary school or incomplete high school	16	24.2	22	32.8	
Complete high school or incomplete higher education	45	68.2	39	58.2	
Complete higher education	1	1.5	3	4.5	
**Occupation**					
House chores	37	56.1	35	52.3	0.772*
Paid work	21	31.8	21	31.3	
Student	8	12.1	11	16.4	
**Marital status**					
With a partner	43	80.3	61	91.0	0.078*
Without a partner	13	19.7	6	9.0	
**Lives with partner**					
Yes	49	74.2	52	77.6	0.651*
No	17	25.8	15	22.4	
**Previous disease**					
Yes	1	1.5	3	4.5	0.319*
No	65	98.5	64	95.5	
**First-degree laceration**					
Yes	45	68.2	37	55.2	0.126*
No	21	31.8	30	44.8	
**Second-degree laceration**					
Yes	31	47.0	28	41.8	0.549*
No	35	53.0	39	58.2	
**Episiotomy**					
Yes	2	3.0	6	9.0	0.152*
No	64	97.0	61	91.0	
	***Mean***	***SD***	***Mean***	***SD***	***p***
	n=65		n=65		
**Maternal age** (years)	21.3	4.3	21.9	4.8	0.745^†^
	n=65		n=64		
**Gestational age** (weeks)	39.6	1.1	39.6	1.1	0.761^†^
	n=65		n=63		
**Body mass index** (kg/m^2^)	27.3	4.3	28.1	4.2	0.312^‡^

* Chi-squared test.

† Wilcoxon-Mann-Whitney test.

‡ t-test.

There were no significant differences in UI regarding the type of repair, although there was a significant variation considering the gestational and postpartum periods (p=0.031). In both study groups, the frequency of UI was higher during pregnancy and decreased up to 6–8 postpartum months; however, its prevalence increased two months after birth, regardless of the type of repair (Table 2).

**Table 2:** Urinary incontinence (UI) at different stages among primiparous women undergoing perineal repair during birth with surgical glue or sutures, according to the type of perineal repair, RCT follow-up, São Paulo, Brazil, 2017–2018 (N=133)

*Stage*	*Total n*	*Perineal repair*	*With UI*		*Without UI*	
			*n*	*%*	*n*	*%*
**Gestation**	66	Glue	12	18.2	54	81.8
	67	Suture	7	10.4	60	89.6
**10–20 days postpartum**	55	Glue	2	3.6	53	96.4
	56	Suture	2	3.6	54	96.4
**50–70 days postpartum**	59	Glue	7	11.7	52	88.3
	62	Suture	4	6.5	58	93.5
**6–8 months postpartum**	20	Glue	1	5.0	19	95.0
	34	Suture	1	2.9	33	97.1

Binomial GLMM. Type of repair: p=0.280. Study stages: p=0.031. Type of repair vs Stages: p=0.953.

Women’s satisfaction levels were evaluated across all three postpartum stages, revealing that the vast majority reported being satisfied or very satisfied, with no significant differences based on the type of perineal repair. The percentage of women reporting being very dissatisfied was at 5.3% between 10 and 20 days postpartum and 3.7% between 6 and 8 months, exclusively among those with sutures. Conversely, the highest satisfaction levels were reported by women evaluated at two months postpartum (p=0.019), irrespective of the type of repair (Table 3).

**Table 3:** Satisfaction levels among primiparous women undergoing perineal repair during birth with surgical glue or sutures at different cohort stages, according to the type of repair, RCT follow-up, São Paulo, Brazil, 2017–2018 (N=105)

*Postpartum stage*	*Satisfaction*	*Perineal repair*			
		*Glue*		*Suture*	
		*n*	*%*	*n*	*%*
**10–20 days** (N=105)		48	100	57	100
	Very satisfied	18	37.5	29	50.8
	Satisfied	27	56.2	20	35.1
	Indifferent	3	6.3	3	5.3
	Dissatisfied	0	0	2	3.5
	Very dissatisfied	0	0	3	5.3
**50–70 days** (N=104)		49	100	55	100
	Very satisfied	24	49.0	32	58.2
	Satisfied	25	51.0	22	40.0
	Dissatisfied	0	0	1	1.8
**6–8 months** (N=51)		24	100	27	100
	Very satisfied	10	41.6	15	55.6
	Satisfied	13	54.2	10	37.0
	Indifferent	1	4.2	1	3.7
	Very dissatisfied	0	0	1	3.7

Cumulative binomial GLMM. Type of repair: p=0.496. Study stages: p=0.019. Type of repair vs Stages: p=0.993.

Table 4 presents the PFMS analysis conducted on 30 women between 6 and 8 months postpartum. The glue group exhibited a PFMS of 32.4 cmH_2_O, compared to 27.4 cmH_2_O in the suture group, with the group that used the glue presenting 5.0 cmH_2_O stronger. However, this difference did not reach statistical significance. Additionally, there were no significant variations in PFMS related to the type of perineal trauma (second-degree laceration and episiotomy).

**Table 4:** Pelvic floor muscle strength (PFMS) among primiparous women according to the type of perineal repair and perineal trauma, 6 up to 8 months after birth, RCT follow-up, São Paulo, Brazil, 2017–2018 (N=30)

*Variable*	*n*	*PFMS (cmH_2_O)*						*p*
		*Mean*	*SD*	*95% CI*	*Median*	*Min*	*Max*	
**Perineal repair**								0.331*
Glue	11	32.4	14.0	23.0–41.8	31.7	14.3	54.4	
Suture	19	27.4	12.8	21.3–33.6	24.2	10.5	50.2	
**2nd degree laceration or episiotomy**								0.792^†^
Yes	12	30.1	13.1	21.7–38.4	28.5	11.0	54.4	
No	18	28.7	13.6	21.9–35.5	24.8	10.5	50.2	

* Wilcoxon-Mann-Whitney test;

† t-test.

The mean values in all FSFI domains were higher among the women in the glue group, and the total score mean was 2.7 points higher, although not statistically significant (Table 5).

**Table 5:** Domains of the Female Sexual Function Index (FSFI) among primiparous women according to the type of perineal repair, 6 up to 8 months after birth, RCT follow-up, São Paulo, Brazil, 2017–2018 (N=30)

*Domain*	*Perineal repair*	*n*	*FSFI score*						p*
			*Mean*	*SD*	*95% CI*	*Median*	*Min*	*Max*	
**Desire**	Glue	11	3.9	0.8	3.3–4.5	4.2	2.4	4.8	0.964
	Suture	19	3.7	1.3	3.0–4.5	4.2	1.2	5.4	
**Arousal**	Glue	11	4.3	1.0	3.6–5.0	4.5	2.4	5.4	0.280
	Suture	19	3.8	1.3	3.5–4.5	3.9	0.0	5.7	
**Lubrification**	Glue	11	4.6	1.2	3.6–5.6	4.8	2.4	6.0	0.828
	Suture	19	4.2	1.7	3.8–5.3	4.2	0.0	6.0	
**Orgasm**	Glue	11	4.6	1.2	3.6–5.8	4.4	2.8	6.0	0.329
	Suture	19	4.0	1.8	3.6–5.2	4.4	0.0	6.0	
**Satisfaction**	Glue	11	5.1	1.0	4.4–5.8	5.2	3.6	6.0	0.756
	Suture	19	4.6	1.7	3.6–5.6	5.2	0.8	6.0	
**Pain**	Glue	11	4.8	1.8	3.2–6.0	5.6	0.4	6.0	0.115
	Suture	19	4.1	1.7	3.6–5.0	4.8	0.0	6.0	
**Total**	Glue	11	27.2	5.3	23.4–31.2	29.0	20.0	33.0	0.504
	Suture	19	24.5	8.6	20.5–29.3	27.4	2.0	33.9	

* Mann-Whitney test.

## DISCUSSION

Both types of perineal repair (surgical glue and polyglycolic acid 910 thread) presented good results concerning the outcomes assessed. However, it is important to consider that the Glubran-2^®^ surgical glue was compared to a fast-absorbing suture thread (Vicryl Rapid^®^), adopting a continuous suture technique, which is considered the gold standard for perineal repair^20,21^ even though this material and technique are not the most utilized in the country.

This study did not identify a significant difference concerning the type of repair and UI. The slight increase in UI between 50 and 70 days postpartum in relation to the UI in pregnancy may be due to significant changes in the woman’s pelvic floor at three months postpartum, such as increased bladder descent at rest and during the Valsalva maneuver^22^. However, in both groups, there was a significant decrease in UI at 6 to 8 months of postpartum compared to the occurrence during pregnancy, as related to the literature^1,8^. These results are consistent with previous studies, on what all women who reported postpartum UI previously or during pregnancy^1^ and indicated that 11.3% and 6.9% of women had UI at 3 and 6 months postpartum, respectively^23^. Also, a study that evaluated two perineal repair techniques (continuous or interrupted) reported an increase in the occurrence of UI at three months postpartum^24^. Regarding surgical glue and UI, it is impossible to compare the data obtained in the present study because, to date, no study has verified the occurrence of UI postpartum with surgical glue.

Additionally, the relationship between perineal trauma and postpartum psychological outcomes, such as depression, anxiety, and post-traumatic stress symptoms, has been explored, and it suggests a significant association between them. As future perspectives of research and clinical care, these results reinforce the importance of comprehensive perineal care and mental health support for postpartum women, highlighting the complex interplay between physical and psychological recovery after childbirth^25^.

In the same way, women’s satisfaction with perineal repair, PFMS, and sexual function the first eight months after birth were consistent.

Women’s satisfaction with perineal repair showed no significant difference between the surgical glue or suture thread of perineal repair. However, all women who reported being very dissatisfied were from the suture group. The reasons for this dissatisfaction have not been discussed with the women. A study conducted with 135 women compared the repercussions of perineal repair using surgical glue and conventional sutures during postpartum and found no statistically significant difference in women’s satisfaction between the groups at all four analyzed moments (day 1, day 2, day 7, and day 30 after birth)^26^. Similarly, another study^12^ did not find significant differences between the types of perineal repair. Nevertheless, a study conducted with 126 women verified greater satisfaction among the women who underwent perineal repair with surgical glue compared to those not subjected to sutures^13^.

Although higher means were verified in the glue group regarding PFMS and sexual function, it is impossible to infer that this type of repair offers better outcomes when compared to the suture group, perhaps due to the small sample size. There is a lack of studies in the literature on these variables comparing both types of perineal repair, which accounts for the novelty of this study. The mean PFMS values found in this study (glue group: 32.4 cmH_2_O, and suture group: 27.4 cmH_2_O) were higher than those reported in the literature for women who experienced perineal trauma (26.6 cmH_2_O and 22.0 cmH_2_O, respectively). Moreover, the glue group approached the value found in women without perineal trauma during childbirth (35.5 cmH_2_O)^27^. However, this study found no significant difference between the analyzed groups. Another parameter associated with PFMS is women’s parity, with nulliparous women presenting the highest mean pelvic muscle strength values. Also, no significant differences were observed between women who had normal births without episiotomy or cesarean section^28,29^. Therefore, based on the average PFMS found in this study, it can be inferred that surgical glue achieved good results.

Concerning sexual function measured by the FSFI instrument, although no significance was observed between groups, the mean value found in the women subjected to repair with surgical glue was higher than in those undergoing suture thread repair, with a 2.7-point difference between the glue group (mean of 27.2) and the suture group (mean of 24.5). The results in the glue group show that the mean FSFI score is not consistent with sexual dysfunction, given that <26.5 points is the cutoff value to identify the presence of sexual dysfunction, according to the literature^30,31^. A cross-sectional study comparing the sexual function between 45 and 180 days after birth related to the type of birth among 85 women revealed that sexual dysfunction occurred in 78.2% of them. The women who underwent vaginal birth had a total FSFI score of 22.2 points, compared to 21.1 points for those who underwent cesarean sections^32^. The current study verified that the lowest score across the glue and suture groups was in the ‘Desire’ domain, followed by the ‘Arousal’ domain of the FSFI. This finding is similar to that of other studies that also reported a lower score in the sexual desire domain^30,32^. Related to the ‘Pain’ domain assessed by FSFI, this study showed a lower pain score between 6 and 8 months after birth among women who had the perineal trauma repaired with surgical glue compared to those who underwent suture thread repair. Several studies indicate that perineal pain and dyspareunia resulting from perineal trauma after normal birth are considered the main factors that affect sexual activity after childbirth, emphasizing the importance of minimizing perineal harm^30^.

### Strengths and limitations

Considering the gap in scientific knowledge regarding the use of surgical glue to repair perineal traumas, the current study presents a potential innovation in comparing various postpartum perineal outcomes with types of perineal repair, a topic that healthcare providers often overlook. Another strength to note is the extended monitoring period, considering its importance in assessing multiple outcomes in a study.

Regarding the study limitations, it is worth mentioning its reduced sample size, resulting from follow-up losses. Between 6 to 8 months after birth, the women participating in the study reported feeling well and not needing to be examined. Even though home visits and telephone contact were offered, we still had many losses, so it was impossible to continue evaluating these women. Therefore, it was not possible to make other associations regarding the type of perineal trauma in the assessments that might be employed in multivariate analyses. The type of perineal trauma (first- or second-degree laceration and episiotomy), although we tried to address it through statistical analysis tests, can be considered as a potential confounder, with a possible modifying effect on the outcomes.

### Implications

Among the main implications for the practice derived from the current study, the use of a less invasive alternative method for perineal repair stands out, which contributes various advantages for professionals and treated women alike, with results that are similar to, or even better, than those obtained with the standard perineal care most indicated by the evidence. The impact of normal birth on women’s perineal conditions and sexual function was emphasized by the current study. It emerged as an area requiring much attention to promote comprehensive and humanized assistance in the postnatal period.

Surgical glue showed good aesthetic and functional results in the perineum at eight months postpartum, representing a viable option for repairing perineal trauma. This study may contribute to strategies for counseling women about perineal care and sexuality in the postpartum period. Surgical glue appears to be a safe and effective alternative to sutures for repair of perineal trauma in vaginal birth. This work signals the need for future research on aspects of the postpartum period that remain underexplored.

## CONCLUSIONS

No significant difference was observed between the type of perineal repair in UI incidence or women’s satisfaction. The PFMS and sexual function scores were consistently higher in the glue group than in the suture group during the first eight months after perineal repair. However, this difference did not reach statistical significance. Surgical glue showed good aesthetic and functional results in the perineum at eight months postpartum.

## Data Availability

The data supporting this research are available from the authors on reasonable request.
